# SCreg: a registration-based platform to compare unicondylar knee arthroplasty SPECT/CT scans

**DOI:** 10.1186/s12891-020-3185-9

**Published:** 2020-03-12

**Authors:** Félix Dandois, Stijn De Buck, Lucas Beckers, Darshan Shah, Laura Slane, Hilde Vandenneucker, Lennart Scheys

**Affiliations:** 1 Development and Regeneration Department, Institute for Orthopaedic Research and Training, KU Leuven, 49 Herestraat – Box 805, 3000 Leuven, Belgium; 2 Radiology and Cardiology Department, University Hospitals Leuven, 49 Herestraat, 3000 Leuven, Belgium; 3 Electrical Engineering Department, KU Leuven, 49 Herestraat, 3000 Leuven, Belgium; 4 Orthopaedics Department, University Hospitals Leuven, 49 Herestraat, 3000 Leuven, Belgium; 5 Mechanical Engineering Department, University of Rochester, 500 Joseph C. Wilson Blvd, Rochester, NY 14602 USA

**Keywords:** Dice similarity coefficient, Osteoblastic activity, Registration, SPECT/CT, UKA

## Abstract

**Background:**

A combination of conventional computed tomography and single photon emitted computed tomography (SPECT/CT) provides simultaneous data on the intensity and location of osteoblastic activity. Currently, since SPECT/CT scans are not spatially aligned, scans following knee arthroplasty are compared by extracting average and maximal values of osteoblastic activity intensity from large subregions of the structure of interest, which leads to a loss of resolution, and hence, information. Therefore, this paper describes the SPECT/CT registration platform (SCreg) based on the principle of image registration to spatially align SPECT/CT scans following unicondylar knee arthroplasty (UKA) and allow full resolution intra-subject and inter-subject comparisons.

**Methods:**

SPECT-CT scans of 20 patients were acquired before and 1 year after UKA. Firstly, scans were pre-processed to account for differences in voxel sizes and divided in volumes of interest. This was followed by optimization of registration parameters according to their volumetric agreement, and alignment using a combination of rigid, affine and non-rigid registration. Finally, radiotracer uptakes were normalized, and differences between pre-operative and post-operative activity were computed for each voxel. Wilcoxon signed rank sum test was performed to compare Dice similarity coefficients pre- and post-registration.

**Results:**

Qualitative and quantitative validation of the platform assessing the correct alignment of SPECT/CT scans resulted in Dice similarity coefficient values over 80% and distances between predefined anatomical landmarks below the fixed threshold of (2;2;0) voxels. Locations of increased and decreased osteoblastic activity obtained during comparisons of osteoblastic activity before and after UKA were mainly consistent with literature.

**Conclusions:**

Thus, a full resolution comparison performed on the platform could assist surgeons and engineers in optimizing surgical parameters in view of bone remodeling, thereby improving UKA survivorship.

## Background

Despite the numerous advantages of unicondylar knee arthroplasty (UKA) compared to total knee arthroplasty [1–5] and its increasing use in clinical practice (+ 32.5% per year in US [6]), this procedure has still a lower survivorship at long term (70% vs 89% at 15 years [7]). The main causes explaining this low survivorship are aseptic loosening and osteoarthritis progression [8] which can both be linked to bone remodeling [9–13]. Therefore, an indicator based on bone remodeling [9, 10, 14–16] could be greatly beneficial to improve UKA survivorship. In order to characterize bone remodeling, spatial patterns and temporal evolutions of osteoblastic activity, which is representative of bone remodeling following arthroplasty, can be observed.

SPECT/CT is the modality typically used to measure those spatiotemporal changes in osteoblastic activity. SPECT, or single photon emission computed tomography, can quantify the intensity of osteoblastic activity [17] and its combination with CT, or computed tomography, provides the documentation of the anatomic location of the activity.

However, in order to observe changes in osteoblastic differences through time or between different surgical parameters, SPECT/CT scans must be compared both longitudinally, i.e. at different timepoints for a same patient, and/or transversely, i.e. between different patients.

Nevertheless, due to differences in patient positioning within the scanner or anthropometric differences between patients, SPECT/CT scans are usually not mutually aligned and thus cannot be directly compared. To overcome this challenge, regional classification systems, in which several regions are manually defined based on anatomic landmarks and/or predefined dimensions, have been developed for evaluating unicondylar knee [14, 18], total knee [9, 10, 19], total hip [20, 21] and shoulder [22] arthroplasty. Despite prior success with this approach, it is limited in that it significantly reduces data resolution as thousands of SPECT voxels are reduced into just a few regions for analysis (around 200 voxels per region) leading to the loss of local information. Therefore, full resolution comparisons are required to avoid any loss of information with the potential to give better insight in the evolution of osteoblastic activity following UKA.

By consequence, an image registration problem [23–25] must be solved in order to spatially align SPECT/CT scans and enable full resolution comparisons. Considering the differences in patient positioning during SPECT/CT acquisitions, several approaches have been proposed in the literature to solve this problem – for instance, standalone rigid transformations [26], global scaling [27], and the use of external markers to combine these approaches [28, 29]. Despite their success in intra-subject registration of hard tissues, such as bone, these procedures cannot tackle inter-subject alignment, since they do not consider anthropometric variability. Non-rigid transformations, which could overcome this issue, have only been used to further optimize intra-subject alignment of SPECT/CT scans [30–32]. However, to enable an accurate inter-subject alignment necessary to perform transverse analysis of SPECT/CT scans, variability in the joint condition, either due to anthropometry or due to surgical intervention such as arthroplasty must be taken into account. Indeed, this variability causes the registration problem to be more challenging to solve and, to our knowledge, there is no reported study in the literature discussing registration procedures to align hybrid SPECT/CT scans both within and between UKA subjects.

Therefore, the aim of this study is to develop and validate a platform, further referred as SCreg (standing for SPECT/CT registration platform), to spatially align hybrid SPECT/CT scans of UKA patients with the potential to allow full-resolution intra-subject and inter-subject comparisons of osteoblastic activity patterns using a combination of rigid, affine and non-rigid transformations.

## Methods

### Data collection

Following receipt of consent form from the patients, hybrid SPECT/CT images in dcm format of 20 patients (Age: 60 ± 10; BMI: 29 ± 5 kg/m^2^; Gender: 15 males, five females) slated to receive a medial UKA prosthesis (Oxford partial knee, Zimmer-Biomet, Warsaw, USA) were acquired before surgery (20 ± 12 days) and one year following surgery (369 ± 16 days). All surgeries were carried out by the same surgical team. All scans were carried out with a hybrid system (Symbia T16; Siemens, GmbH, Germany) equipped with a dual-head gamma camera and an integrated 16-slice CT scanner (Collimation of 16 × 0.75 mm). Limbs were placed in a supine position and patients were specifically told to stay as immobile as possible under supervision of a licensed technician according to standard scanning protocol. SPECT acquisitions were performed in the late delayed metabolic phase, 2 h following the injection of a 750 MBq dose of ^99^mTechnetium-hydroxymethane diphosphonate isotope (HDP; Curium, Brussels, Belgium). SPECT was set to a matrix size of 128 × 128 with a zoom of 1, an angle step of 32, a time per frame of 16 s and 180 degrees orbit. Subsequently, a high-dose CT was carried out, scans were then reconstructed with a metal artefact reduction protocol. Final SPECT scans had a voxel size of (x/y/z) 4.79/4.79/4.79 m, while CT scans had a voxel size of either 0.98/0.98/3 mm or 1.27/1.27/3 mm. Lower CT resolution was used for pre-operative scans of three patients and post-operative scans of six patients.

### Data processing

**Fig. 1:**
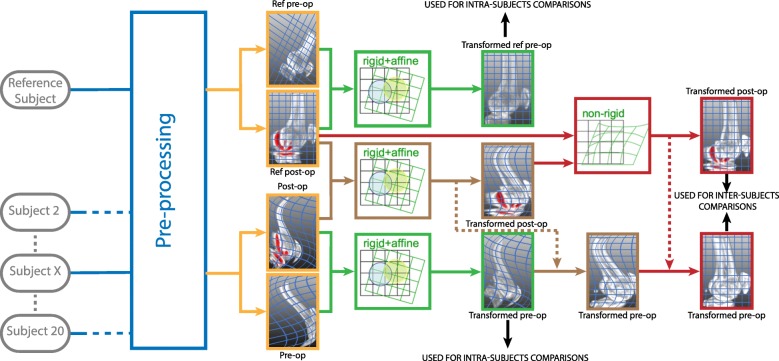
Schematic representation of the registration platform workflow. Legend: All scans (grey) are first pre-processed (blue). The images obtained (orange) are then registered. Affine registration (green) is used to align intra-subjects scans and a combination of rigid (brown) and non-rigid (red) registration is used for inter-subjects scans alignments. Once aligned, SPECT is analyzed and intra- and inter-subjects comparisons are performed

Throughout this paper, the coordinate system used, x, y and z are parallel to the patient’s frontal, sagittal and transverse axis respectively. The SPECT/CT scans were subjected to three operations within SCreg, all performed in MeVisLab (MeVis Medical Solutions AG, Germany)– pre-processing, registration and SPECT-based statistical analysis of osteoblastic activity (Fig. 1). Each SPECT/CT scan had a common coordinate system for both imaging modalities; hence, same transformations were applied to SPECT scans during registration as those applied to the associated CT scan, without any disruptions in the alignment.

To account for resolution differences between image datasets, all CT and SPECT scans were resampled to a voxel size of 0.98/0.98/3 mm. Right legs were reflected about the sagittal plane to obtain a ‘left’ laterality for all specimens. To consider all possible changes in joint position, images were divided in three volumes of interest (VOIs) – femur, tibia and patella.

The cuboidal VOIs were defined in CT space using bony landmarks shown to be accurately identifiable [33] as well as other distinctive bony landmarks previously described [34] on the femur (the fibula head, the femoral trochlea proximal (FTP), the femoral lateral epicondyle (FLE) and the femoral medial epicondyle (FME)), tibia (femoral knee center (FKC) and tibia lateral and medial peaks) and patella (proximal, distal, lateral and medial patella margins). Within each VOI, an intensity threshold was used not only to isolate the bony tissue, but also to mask the prosthesis in post-operative scans thereby avoiding complications during registration.

The registration process was developed using the Elastix toolbox [25, 32, 35, 36], developed specifically to perform image registration. However, despite including all the required parameters, their values must still be defined by the user. To account for differences in positioning inside the scanner, an affine registration (AR) was used for intra-subject registration. In contrast, inter-subject registration, which required the consideration of additional anthropomorphic differences, involved AR followed by non-rigid registration (NRR) with additional non-affine transformations. Registration parameters (Table 1) were tuned by registering five randomly selected femoral bone images with typical values from literature [25, 32, 35]. The selected parameters were the one preserving the image integrity upon visual inspection and maximizing the Dice similarity coefficient (DSC) [25, 35, 37, 38].

**Table 1:** Values of main registration components following parameters tuning for affine and non-rigid registration

Main parameters	Affine registration	Non-rigid registration
**Pyramids**	Smoothing anisotropic gaussian	Smoothing anisotropic gaussian
**Number of resolutions**	3	4
**Smoothing factors**	442,221,111	884, 442, 221, 111
**Metrics**	Normalized mutual information	Non-normalized mutual information
**Number of histogram bins**	16	32
**Transforms**	Affine	B-Splines
**Control grid spacing**	Not applicable	(32; 32; 16) voxels
**Multiplication factors**	Not applicable	884 442,221,111
**Optimizers**	Adaptive stochastic gradient descent	Adaptive stochastic gradient descent
**Maximal number of iterations**	2000	500
**Samplers**	Random	Random
**Number of points**	2000	2000
**Interpolators**	Nearest neighbor	Linear
**Final interpolators**	Linear	3rd-degree B-splines

For intra-subject comparison, registration was performed between images of the same patient but at different timepoints. The reference image, further referred as fixed image, was the post-operative scan, with masked-out prosthesis, and the image transformed to the fixed image, further referred as the moving image, was the pre-operative one. For the inter-subject registration, the fixed image was the post-operative bone image of a reference subject randomly selected amongst the 14 post-operative scans with a higher resolution and the moving image was the post-operative bone image of any other subject. Prosthesis masks were not required for inter-subject registrations, since the same prosthesis was used for all subjects, hence no intrinsic significant differences requiring a masking. In addition, it allows to take into account the extrinsic differences in terms of implantation. The transformations from inter-subject registration were concatenated with those from the intra-subject registration.

To increase the chances of successful registration, a multiresolution approach, aimed at simplifying the data by iterative image smoothing, was implemented [35]. This approach registered large and dominant structures before moving on to progressively smaller structures. Three resolution stages were implemented for AR, and four for NRR, using a common gaussian pyramid blurring the image. Smoothing factors were defined for every stage and direction (Table 1), with each factor representing lowered resolution in the x, y and z directions of the images respectively.

A B-Spline representation [39], modeled as a weighted sum of B-Spline basis functions placed on a uniform control grid, was used to define NRR for inter-subject registration. The resolution of this control grid defined the flexibility of the transformations, starting with a larger grid for the first resolution stage and progressively decreasing to end with a thinner grid, allowing for more deformation of the image, for the last resolution stage.

Normalized mutual information [40, 41] was chosen as the metric for image similarity. This metric required the computation of the joint histogram based on the dynamic range of the images. Following parameter tuning, 16 bins were defined. For NRR, since the presence of the prosthesis increased the dynamic intensity range of the images, the number of histogram bins was greater than that used for AR.

To find the optimum of the mutual information-based cost function, an iterative optimization was performed using a robust and adaptive stochastic gradient descent method (Robbins-Monro) [42, 43]. This method reduced the computation time by using a small subset of points from the fixed image at each iteration to compute the derivative of the cost function. Maximal number of iterations was used as a stop criteria for the optimization since it is the only criteria available in Elastix and its value for AR and NRR was defined during parameter tuning.

A sampler was used to select the subset of points required for the optimizer. It involved a random selection of points at each iteration for the stochastic optimization, here based on the Halton sampling, thereby improving the smoothness of the cost-function and avoiding two known problems related to the use of mutual information, “overlap problem” and “grid effect” [44].

Interpolation of the gray values between voxels was required owing to the selection of random points by the sampler, which did not necessarily ensure that the corresponding points in the moving image were at a voxel position. In case of the AR, nearest neighbor interpolation was selected, with a linear interpolator to generate the last deformed image [29], while for the NRR, a linear interpolator was selected, with a third degree B-spline interpolator to generate the last deformed image.

Once scans were registered, tracer uptakes were normalized to account for subject-specific differences in tracer metabolization. For each scan, average osteoblastic activity calculated within a rectangular volume of 6008.5 mm^3^ (2100 voxels) at a distance of 10 cm from the FKC along the z axis was used for normalization. Pre-operative osteoblastic activity was subtracted from post-operative values for each voxel, and an aggregate map was created by averaging differences within each voxel value over all co-registered subjects, thereby providing a unique representation of all the subjects.

### Validation

Since no unique tool allows the complete validation of a registration [35], multiple methods were employed, including qualitative criteria for preliminary evaluation and quantitative criteria for objective evaluation. The registration was considered successful only if all criteria were fulfilled.

All images were first qualitatively evaluated for image integrity, including the presence of image blur, irregularities, such as holes, and loss of bone contour shape. This was followed by qualitative assessment of the conformance of bone contours on a two dimensional overlay of the two registered images over a few horizontal slices of CT selected in the region with the prosthesis, since this region was expected to be exposed to the most errors in registration.

Following qualitative assessment, volumetric agreement between the registered images was quantified by computing the DSC and used as quantitative validation. Considering the resolution of the CT and SPECT images, complexity of the structures, and the presence of an implant, a DSC over 80% was defined as the criterion for successful registrations [25]. In addition, DSC were also acquired before and after intra-subject and inter-subject registration, in order to observe if registration significantly improved spatial alignment. Since the data did not follow a normal distribution, as assessed by a Kolmogorov-Smirnov test of normality, a Wilcoxon signed rank sum test (Matlab R2016, MathWorks Inc., Natick, USA) was performed to analyze the effect of registration on DSC, and a Mann Whitney U test was applied to evaluate the impact of varying resolution of pre-operative and post-operative CT images on DSC. *P*-values below 0.05 were considered statistically significant.

The difference between coordinates of anatomical landmarks in registered images was used as the second criterion for quantitative validation. Considering the ratio of the SPECT resolution over the CT resolution, a difference lower or equal to (2;2;0) voxels was considered as satisfactory to assure that the registered points were associated to the same SPECT voxel.

Osteoblastic activity was used as the final criterion for quantitative validation. Apart from comparisons with the literature [14], the location of main increased activity on the aggregate map was also compared with an ongoing study on a superset of the population used in this project and performed by one trained blinded nuclear radiologist using the regional classification system for analysis [18]. In addition, since osteoblastic activity is directly linked to bone strain as explained by the Wolff’s law [45], osteoblastic activity was compared with the location of increased bone strain following UKA [46].

## Results

Qualitative validation showcased successful registrations for all subjects with the absence of image blur, irregularities and loss of bone contours.

Registration resulted in significantly improved DSC values greater than 80% for all VOIs defined previously (Table 2). Different resolutions of pre-operative and post-operative CT images revealed a significant impact on DSC following intra-subject registration. Average distances between the coordinates of selected landmarks following intra-subject and inter-subjects registrations were within the satisfactory limit (Table 3).

**Table 2:** Average DSC pre- and post-registration and associated *p*-values

	Pre-registration (mean ± std in %)	Post-registration (mean ± std in %)	***P***-values (pre to post)	***P***-values (different resolutions)
**Intra-subjects**	75.00 ± 8.49	87.08 ± 3.18	< 0.001	0.0279
**Inter-subjects**	72.63 ± 6.05	83.93 ± 1.85	< 0.001	0.0649

**Table 3:** Average coordinate differences in voxels between specific landmarks along the three axes

	Landmarks	x axis	y axis	z axis
**Intra-subjects**	**FKC**	0.75	0.4	0
	**FME**	0.55	0.5	0
	**FLE**	0.35	0.35	0
	**FTP**	0.4	0.65	0
**Inter-subjects**	**FKC**	1.63	1.13	0
	**FME**	1	1.19	0
	**FLE**	1.06	1.29	0
	**FTP**	1.18	1.24	0

**Fig. 2:**
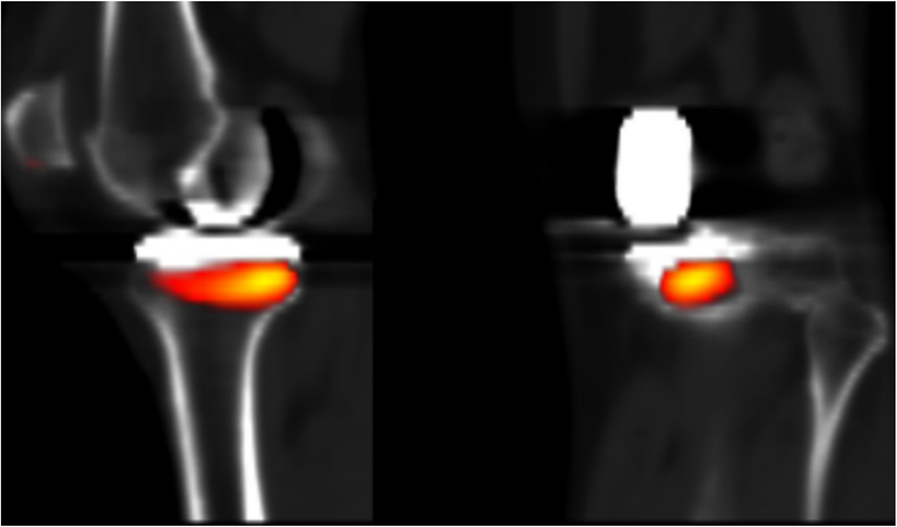
Average increased SPECT activity overlaid onto reference CT. Legend: Left: sagittal view, Right: frontal view. Intensity ranging from red (low activity) to yellow (high activity)

The areas of increased osteoblastic activity on the aggregate map were located below the tibial insert in the posterolateral position (Fig. 2) and on the medial patella facet. A decrease of the activity was observed in the rest of the bone.

## Discussion

The in-house SCreg successfully performed intra-subject and inter-subject registration of CT images from subjects undergoing UKA, thereby allowing full-resolution comparisons, as well as identification of post-surgical rise in SPECT activity. To our knowledge, this is the first study presenting a complete pipeline for registration and analysis of UKA SPECT/CT scans intra- and inter-subjects including the additional steps necessary to go from raw images to the SPECT analysis of a population. Indeed, in addition to the tuning of the parameters of the Elastix toolbox, pre-processing and analysis tools as well as the complete workflow were described. The pre-processing step allowing a normalization and optimization of the scans prior the registration while limiting the required user inputs and the post-processing providing analysis tools to summarize an entire population data into one single representation. However, as it will be discussed hereunder, each part of the platform is a compromise between quality, time and user input in order to have the most suitable application for the clinic environment.

For intra-subject registration, the use of affine transformation greatly limits the possibility of image blur and holes within the image insuring a conservation of image integrity. Concerning the inter-subject registration, the higher magnitude of deformations inherent to the use of non-linear transformations could have led to invalid results. However, the use of an affine registration prior to the non-rigid one, the correct parametrization of the control grid for the B-spline transformations and the hierarchical approach allowed to preserve image integrity.

DSC values, although satisfactory post-registration (Table 2), have the potential to be further improved. In the case of intra-subject registrations, the use of additional non-linear transformations could improve the results; however, this would increase the processing time and the risk of image degradation. In the case of inter-subject registration, a thinner grid could allow more flexibility to the non-linear transformations, thereby improving DSC values. Although, parameter tuning revealed a higher risk of degrading the images during this implementation. It should also be noted that inter-subject registrations were performed using a reference subject randomly selected and results could have been improved if the selected reference subject was the one with the least differences with respect to the other subjects. However, satisfactory DSC despite the random selection of the reference scan demonstrate the robustness of the process.

Significant differences in DSC values were obtained for the intra-subject registration performed on CT images with different resolutions, but not for the inter-subject registration. This could be explained by the use of non-linear transformations in inter-subject registration, which compensates for these differences. However, since this cannot be adopted in intra-subject registration for the aforementioned reasons, it is advisable to acquire the CT images with identical resolution for comparative analysis.

The distance between the coordinates also indicates a successful registration since all values are below (2;2;0) voxels. By consequence, the SPECT voxel associated to a CT voxel on co-registered scans is the same thereby allowing voxel by voxel comparison of SPECT intensities. In addition, the landmarks selected to perform that comparison have an intra-rater and inter-rater reliability below the CT voxel size [33] attesting the robustness and the validity of that measurement.

Concerning the SPECT analysis, aggregate maps created with SCreg were compared with the clinical interpretation of a nuclear radiologist based on the classification system performed on a population including the patients analyzed in this project [18]. Similarly, a general decrease of activity was observed but no increased normalized activity was noticed neither on the tibia nor the patella region. Nonetheless, the tibial area identified in this study as a common region of increased osteoblastic activity correlates with increased strain area following UKA [46]. Bone strain being directly linked to osteoblastic activity through Wolff’s law, it was expected to observe an increase of activity in that region. By consequences, the differences between the two approaches could be explained by the averaging performed within the classification method. Indeed, the areas located in this study could have been separated in the study using the classification scheme hence the increase would have been masked off by the surrounding decrease of activity. These differences suggest that the use of full resolution could provide more insight into bone remodeling following UKA compared to the classification system.

One limitation of this study is the population size (40 scans in total). However, we found it to be similar to the 45 scans used by Papavasileiou et al. [29] (although obtained through simulation for validation purposes) and much higher to other studies using less than 10 scans [28, 30, 31]. However, as a limitation, it must to be noted that all prostheses were identical, thereby limiting the variability of images during registration. Another limitation is the beam hardening artefact caused by the metallic implant during CT scan. Although, the use of a metal artifact reduction algorithm during CT reconstruction limited these artefacts, some streaking artefacts were still visible in some patients; however, these were mitigated by masking the prosthesis during registrations, thereby affecting results minimally.

The main limitation of the platform is linked to the segmentation of the bones in three parts which is done through cuboidal volumes. In this approach, 2000 points are randomly selected within the volume, such that in a case where the volume is large compared with the bone, a large percent of points selected will be in the background and by consequence the quality of the registration will be affected. Therefore, a segmentation approach that limits the image to the actual bone, could improve the registration process. Nevertheless, the approach we used allows to facilitate the bone segmentation and is most suitable for the surgeons which are considered as the main SCreg users in the future. Indeed, the identification of an accurate segmentation, from a technical point-of-view, allowing a smooth registration process would require significant time and a knowledge surgeons might not especially have in comparison to the location of CT landmarks as in the current approach. Alternatively, by using a large number of samples combined with a high number of resolutions, we can address this challenge. Therefore, the simplified approach used in the SCreg reduces computational time and limits required user inputs, thus reducing time and inter-rater differences. In addition, this method increases the intra- and inter-rater reliability since for specific landmarks, it was shown to be lower than the CT voxel size used in this study [33] and for the other landmarks, although not assessed in details, they are easily identifiable on the CT images.

Concerning the future perspectives, an in-depth analysis of the osteoblastic activity changes following UKA in asymptomatic population with SCreg could allow to identify typical patterns. In addition, comparisons with symptomatic population using statistical maps could allow to identify locations with a significantly different osteoblastic activity change following UKA associated with symptoms.

## Conclusions

A registration-based platform was developed and validated to compare pre-operative and post-operative SPECT-CT images within, as well as across, subjects undergoing UKA. SCreg allows full-resolution comparisons without loss of data to the contrary of current analysis methodology. With high efficiency and minimal user input, SCreg could be used by clinicians and/or researchers to improve our knowledge concerning UKA as well as patient’s quality of life. Moreover, with an adaptive algorithm and a generic process pipeline, SCreg is neither limited by the type of surgical intervention, nor by the location in the body.

## Data Availability

The datasets generated during and/or analyzed during the current study are not publicly available due to the fact the data contains sensitive patient information but are available from the corresponding author on reasonable request.

## Abbreviations

UKA: Unicondylar knee arthroplasty
SPECT: Single photon emission computed tomography
CT: Computed tomography
SCreg: SPECT/CT registration platform
VOI: Volume of interest
FTP: Femoral trochlea proximal
FLE: Femoral lateral epicondyle
FME: Femoral medial epicondyle
FKC: Femoral knee center
AR: Affine registration
NRR: Non-rigid registration
DSC: Dice similarity coefficient
